# Virtual Reality Versus Monitor-Based Distraction in Children with Mild Intellectual Disability: A Preliminary Comparative Observational Study

**DOI:** 10.3390/children13030437

**Published:** 2026-03-23

**Authors:** Antonio Fallea, Simone Treccarichi, Simona L’Episcopo, Massimiliano Bartolone, Luigi Vetri, Mirella Vinci, Raffaele Ferri, Francesco Calì

**Affiliations:** 1 Oasi Research Institute-IRCCS, 94018 Troina, Italy; streccarichi@oasi.en.it (S.T.); slepiscopo@oasi.en.it (S.L.); mbartolone@oasi.en.it (M.B.); mvinci@oasi.en.it (M.V.); rferri@oasi.en.it (R.F.); cali@oasi.en.it (F.C.); 2 Child Neuropsychiatry Unit of Nicosia, UOC NPIA-ASP Enna, 94014 Nicosia, Italy; luigi.vetri@asp.enna.it; 3 Department of Medicine and Surgery, Kore University of Enna, 94100 Enna, Italy

**Keywords:** virtual reality, dental treatment completion, neurodevelopmental disorders, corah dental anxiety scale

## Abstract

**Background/Objectives**: Dental anxiety represents a significant barrier to oral care in children with neurodevelopmental disorders (NDDs), whose sensory sensitivities and behavioral challenges often complicate clinical management and limit access to treatment. Virtual reality (VR) has emerged as a supportive tool to improve the feasibility of dental procedures in this vulnerable population. This study aims to evaluate whether a VR-based distraction approach could facilitate the completion of dental treatment in children with mild intellectual disability (ID). **Methods**: A prospective comparative observational study was conducted between February and September 2025 involving 56 children aged 11–15 years with mild ID and moderate dental anxiety (Corah Dental Anxiety Scale, DAS: 9–12). Participants were allocated to two groups of distraction approaches—VR distraction (n = 28) using the Oculus Quest 3^®^ headset or a monitor-based cartoon (n = 28)—according to device availability and to maintain balanced group sizes. The primary outcome was treatment success, defined as completion of the restorative dental procedure under local anesthesia within 50 min. **Results**: Treatment success was achieved in 78.6% of the VR group versus 46.4% of the monitor group (*p* = 0.026). The odds of successful treatment were more than four times higher with VR compared to monitor distraction (OR 4.12; 95% CI: 1.16–16.47), with a risk ratio of 2.50 (95% CI: 1.14–5.50). Stratified analysis suggested a stronger effect in females (OR 12.25; 95% CI: 1.27–118.36) than in males (OR 2.56; 95% CI: 0.53–12.43). **Conclusions**: VR-based distraction significantly improved dental treatment success in children with mild ID compared with conventional distraction. Although gender differences were observed, they should be interpreted with caution due to the small sample size. This work lays the foundation for developing both short- and long-term protocols to facilitate dental treatment management and cooperation in patients with NDDs.

## 1. Introduction

Odontophobia is defined as an extreme and disproportionate fear of dental treatment and of any procedure involving the oral cavity. Individuals affected may avoid dental visits even in the presence of urgent treatment needs [1,2,3]. Dental phobia and dental anxiety often emerge during childhood, and when persistent, they can result in avoidance of dental care, leading to deterioration of oral health and a reduced quality of life. While a certain level of dental fear is considered developmentally normative in children, excessive or persistent fear that progresses into clinically significant dental anxiety or odontophobia can seriously compromise both oral and general health across the lifespan [4,5]. The assessment of patients’ anxiety is crucial for the effective management of dental treatment, particularly in preventing uncooperative behaviors. Anxiety can be quantified using self-report questionnaires, and several validated scales have been developed for this purpose. Among them, the Corah Dental Anxiety Scale (DAS), introduced in 1978, is the most widely adopted [6]. The DAS consists of four multiple-choice items describing common dental situations (e.g., anticipating a dental visit, waiting in the dentist’s office, undergoing drilling or scaling). Each item is rated on a 5-point scale, yielding a total score from 4 to 20. Several modifications of the scale have been proposed; however, the original DAS remains one of the most commonly applied measures [7,8,9].

**Figure 1:**
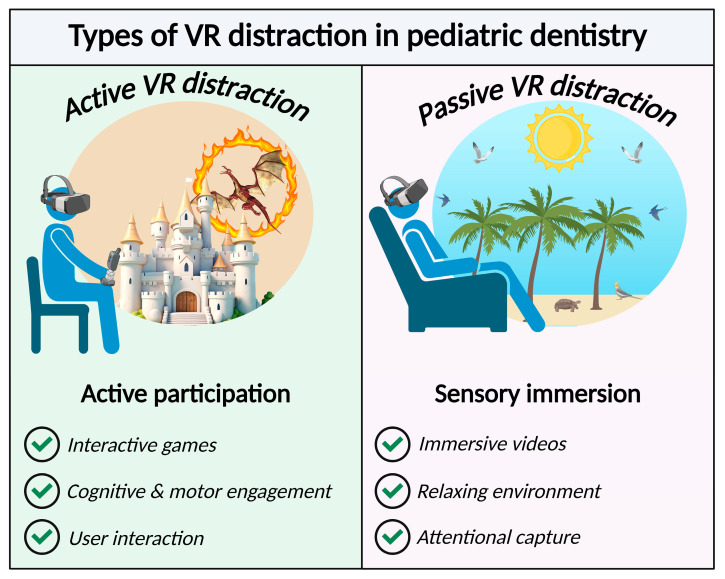
A schematic representation of active and passive VR-based distraction approaches used in pediatric dentistry. Active VR distraction involves direct user interaction with the virtual environment, typically through interactive games or tasks that require cognitive and motor engagement. In contrast, passive VR distraction provides immersive audiovisual stimulation without requiring active participation, exposing the patient to relaxing virtual environments that capture attention and promote sensory immersion during dental procedures.

Dental anxiety is particularly common among individuals with neurodevelopmental disorders (NDDs), such as autism spectrum disorder (ASD), intellectual disability (ID), or borderline intellectual functioning (BIF) [10,11]. Several studies have highlighted the increased need for dental treatment among children with NDDs, mainly due to poor oral hygiene resulting from limited motor skills required for effective plaque removal and a reduced ability to cooperate during dental care [12]. In these populations, anxiety is frequently compounded by behavioral difficulties that make cooperation during dental treatment challenging. They exhibit heightened sensitivity (e.g., to noise, light, touch, or taste), making dental environments overwhelming [12]. Communication and cognitive impairments may hinder understanding of dental procedures, increasing feelings of unpredictability and loss of control. Furthermore, previous negative experiences, difficulty adapting to new situations, and anxiety related to physical restraint or separation from caregivers can exacerbate fear responses. Consequently, dental care for patients with NDDs often represents a substantial burden for both families and clinicians. Distraction techniques used during pediatric dental procedures can be broadly classified as passive or active [13]. Passive distraction involves exposure to audiovisual stimuli without requiring active cognitive or motor engagement from the child, such as watching cartoons or using audiovisual glasses. In contrast, active distraction requires direct interaction with the stimulus and greater attentional involvement, as exemplified by the use of interactive tablets or digital games [13]. Virtual reality (VR)-based distraction can be implemented as either an active or a passive intervention, depending on the level of user interaction required. Active VR involves direct cognitive and/or motor engagement with the virtual environment, such as interactive games or tasks that require user input. In contrast, passive VR provides immersive audiovisual stimulation without requiring active participation, relying on sensory immersion and attentional capture to engage the user. Both approaches have been used in clinical settings, and their effectiveness may vary according to the patient’s age, cognitive abilities, and the clinical context in which VR is applied [14]. Figure 1 schematizes the two main virtual reality-based distraction approaches used in pediatric dentistry: active and passive VR.

In this context, VR has emerged as a promising tool to address dental anxiety. VR offers immersive distraction during potentially stressful procedures and can also serve as an acclimatization and relaxing strategy, helping patients familiarize themselves with the clinical environment [15,16,17,18,19]. By diverting attention away from the dental setting and reducing anticipatory fear, VR may facilitate greater cooperation and improve treatment outcomes, particularly in vulnerable populations such as children with NDDs [20,21,22]. Notably, the interactive nature of VR can capture and maintain the attention of children with mild intellectual disabilities, reducing environmental distractions and enhancing emotional engagement during dental procedures [23]. Moreover, by providing multisensory stimulation and realistic yet controllable scenarios, VR facilitates cognitive processing and adaptive emotional responses, helping children better manage anxiety and cooperate during treatment. Despite growing interest in virtual reality-based distraction during dental procedures, evidence regarding its role in facilitating dental treatment completion in children with mild intellectual disability remains limited.

This study aimed to evaluate the effectiveness of a VR-based distraction approach compared with a cartoon monitor in a cohort of individuals with mild intellectual disability during dental treatment. We hypothesized that there would be a significant difference between the two interventions, thereby rejecting the null hypothesis of no difference in treatment success, defined as completion of the dental restorative treatment within the scheduled time.

## 2. Materials and Methods

### 2.1. Sample Recruitment

In this study, we conducted a prospective comparative observational study between February and September 2025 at the Oasi Research Institute–IRCCS, Troina (EN), Italy. The study protocol was approved by the Local Ethics Committee (“CEL-IRCCS Oasi Maria SS.”; 15 March 2023; approval code: 2023/15/03/CE-IRCCS-OASI/253). Written informed consent was obtained from the parents or legal guardians of all participants prior to enrollment. The study was conducted in accordance with the ethical principles outlined in the Declaration of Helsinki. Eligible participants were recruited among patients referred to the diagnostic and rehabilitation services of the Oasi Research Institute–IRCCS.

At enrollment, all patients completed the Corah Dental Anxiety Scale to assess anxiety levels.

### 2.2. Inclusion and Exclusion Criteria

Inclusion criteria consisted of age between 11 and 15 years, a confirmed diagnosis of mild ID established by neuropsychiatrists according to DSM-5 criteria, moderate anxiety as measured by the Corah test, and the presence of at least one decayed tooth requiring conservative treatment under local anesthesia. Exclusion criteria included the absence of anxiety, mild anxiety, high or severe anxiety, and comorbidity with visual disorders.

Dental anxiety was assessed using the Corah Dental Anxiety Scale [6]. This questionnaire consists of four items, each rated on a 5-point multiple-choice scale, referring to common dental situations and evaluating both anticipatory and treatment-related anxiety. Total scores range from 4 to 20 and are classified into five categories: no anxiety (NA, score = 4), slight anxiety (SLA, score 5–8), moderate anxiety (MA, score 9–12), high anxiety (HA, score 13–14), and severe anxiety (SA, score 15–20). Demographic data were collected and integrated with the information derived from the DAS. To ensure consistency, the same trained examiner administered all structured interviews, following standardized instructions and scoring procedures. The scale was administered through structured interviews to ensure comprehension and reliability of responses, considering the cognitive and communicative limitations of individuals with MID examined. A structured interview format allowed the examiner to clarify questions, adapt the pace of administration, and facilitate accurate reporting of anxiety levels, which might not have been achievable with self-administered questionnaires in this population. The DAS was administered during a prior assessment by an examiner who was not aware of the study hypotheses or the experimental conditions.

Of the 72 patients initially screened, 2 were excluded due to comorbidity with myopathy and ophthalmoplegia (manifesting as diplopia and impaired smooth pursuit), 2 due to absence of anxiety, 2 due to mild anxiety, 6 due to high anxiety, and 4 due to severe anxiety. The final study sample consisted of 56 participants (30 females, 26 males) presenting with moderate anxiety. The sample size of 56 participants was determined based on the availability of eligible patients who met the inclusion criteria during the study period.

Once the study sample was defined, participants were assigned to groups through a non-randomized allocation process determined by equipment availability and clinical scheduling, while maintaining a balanced gender distribution between groups. The first group (n = 28; 13 males, 15 females) underwent dental treatment while using a virtual reality (VR) headset as a distraction tool. The second group (n = 28; 13 males, 15 females) received dental treatment while watching a cartoon projected on a monitor positioned in front of the dental chair.

### 2.3. Description of Dental Procedure

All patients received local plexus anesthesia using a cartridge of articaine with vasoconstrictor. Carious tissue was then removed with a round bur mounted on a dental micromotor, followed by restoration with glass ionomer cement. The duration of each dental procedure did not exceed 50 min. This range of time was chosen to ensure the highest accuracy of treatment, allowing sufficient time for the administration of local anesthesia followed by the completion of the dental restoration. Compliance with the interventions (VR or monitor distraction) was assessed through direct behavioral observation during the dental procedure. The operator monitored the patient’s engagement, attention, and interaction with the assigned intervention throughout the session. All participants were blinded to the study hypotheses, while the assessor who evaluated treatment success remained unaware of the study hypotheses/aims. The assessor’s role was limited to determining whether the dental treatment was completed.

### 2.4. Description of Virtual Reality Approach

During anxiety-inducing dental procedures associated with pain, patients were exposed to REALICA VR software (Realica^®^, Montebelluna, TV, Italy), designed to immerse them fully in an alternative virtual environment for the entire duration of the intervention. This immersive setting provided companionship, support, and distraction, thereby reducing focus on the clinical context and the ongoing dental procedures. Simple interactive elements within the virtual environment further enhanced the distractive effect, promoting patient self-management strategies useful beyond the immediate session. All participants were exposed to the same virtual environment content and did not receive any additional human support, coaching, or coping strategies during the procedure. The virtual content consisted of non-interactive relaxing environments inspired by the four classical elements proposed by Empedocles (fire, air, water, and earth). These environments were designed to provide emotionally engaging audiovisual stimulation without requiring active cognitive or motor participation from the child, consistent with a passive immersive distraction approach. The intervention was delivered through a next-generation virtual reality headset (Meta Quest 3^®^, Meta Platforms Inc., Menlo Park, CA, USA; per-eye resolution: 2064 × 2208 pixels; field of view: ~110°; refresh rate: up to 120 Hz), equipped with advanced environmental and motion-tracking sensors, including two RGB cameras and four infrared cameras. The system operated in stand-alone mode, without the need for cables or external devices, and offered a maximum session duration of 60 min, repeatable in a loop if required. A mirroring function allowed healthcare personnel to monitor the patient’s virtual environment in real time. The effectiveness of the intervention was evaluated based on two categories: completion of the dental procedure (treatment success) and failure to complete the procedure (treatment failure). None of the participants had prior experience with a VR device.

### 2.5. Description of Monitor-Based Approach

**Figure 2:**
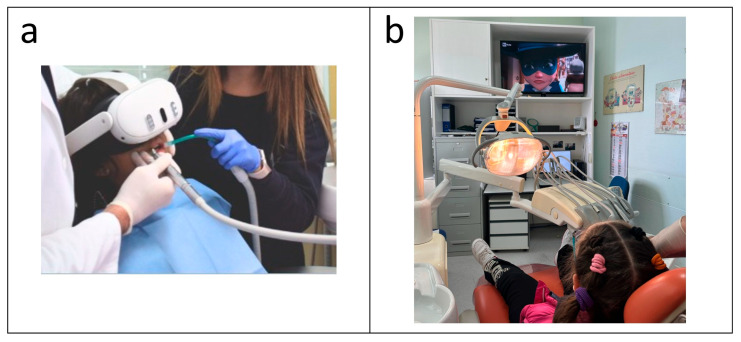
Distraction conditions applied during dental treatment. (**a**) VR-based intervention. (**b**) Conventional distraction method involving a cartoon displayed on a monitor positioned in front of the dental chair.

During dental treatment, patients were invited to watch a cartoon or video of their choice on a monitor positioned in the modified dental operatory. Figure 2 illustrates the two distraction conditions applied during dental treatment.

### 2.6. Data Analysis

Statistical analyses were performed using R studio software version 4.5.2 using the following packages: tidyverse (data manipulation and visualization), readxl (importing Excel files), janitor (data cleaning), epitools (risk ratios and odds ratios), vcd (contingency table analysis and Mantel–Haenszel tests), broom (tidying regression outputs), and gt with gtExtras (tabular reporting).

Descriptive statistics were calculated for demographic and clinical variables. Categorical variables (treatment success vs. failure) were summarized as frequencies and percentages; continuous variables (age and DAS scores) as means ± standard deviations. Comparisons between groups (VR vs. monitor) were performed using the Chi-square test with Yates’ correction or Fisher’s exact test, as appropriate. Measures of effect included odds ratios (ORs) and risk ratios (RRs) with 95% confidence intervals (CIs). The Mantel–Haenszel method was applied to estimate the common OR stratified by gender. Differences in DAS scores between successful and unsuccessful treatments were evaluated using Student’s *t*-test or, when assumptions were not met, the Wilcoxon rank-sum test. A multivariable logistic regression model was fitted to estimate adjusted ORs for treatment success, with group assignment (VR vs. monitor) as the main predictor and age, gender, and DAS score included as covariates. Results were reported as adjusted ORs with 95% CIs. Statistical significance was set at *p* < 0.05 (two-tailed). Post hoc sample size estimation was performed based on the primary binary outcome of treatment success (completion of the dental restorative procedure within the scheduled time). Assuming a two-sided significance level of 0.05 and the observed success rates of 78.6% in the virtual reality group and 46.4% in the monitor group, the analysis was conducted for both 80% and 90% statistical power, with an assumed 10% dropout rate. Power analysis was performed using RStudio (version 4.5.2).

## 3. Results

A total of 56 participants were enrolled in the study, with 28 assigned to the VR group and 28 to the monitor group. The two groups were comparable in age (mean age 12.9 ± 1.5 years in the monitor group; 12.9 ± 1.3 years in the VR group) and baseline anxiety (mean DAS score 10.4 ± 1.1 vs. 10.5 ± 1.2, respectively) (Table 1).

Treatment success was achieved in 22 of 28 patients in the VR group (78.6%) compared with 13 of 28 in the monitor group (46.4%), yielding a difference of 32.1 percentage points (Table 2).

**Figure 3:**
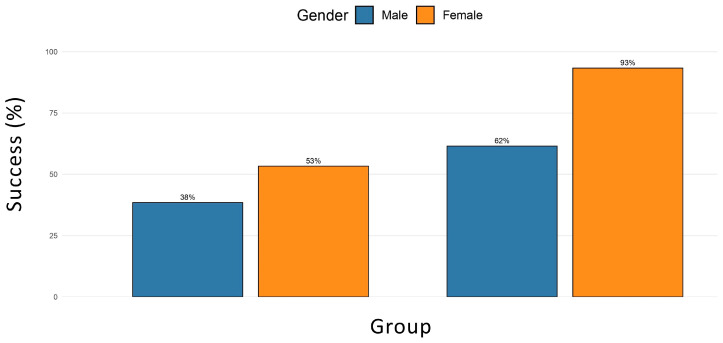
Percentage of treatment success across groups (VR vs. monitor), stratified by gender. Female participants exhibited a markedly higher success rate in the VR group compared with the monitor group.

This difference was statistically significant (χ^2^ = 4.88, *p* = 0.027; Fisher’s exact test *p* = 0.026). The odds ratio for treatment success in the VR group compared with the monitor group was 4.12 (95% CI: 1.16–16.47), and the risk ratio was 2.50 (95% CI: 1.14–5.50). Figure 3 depicts the percentage of treatment success across groups (VR vs. monitor), stratified by gender.

When stratified by gender, the Mantel–Haenszel common odds ratio was 4.66 (95% CI: 1.35–16.09; *p* = 0.026). In males, the association between VR and treatment success was not significant (OR = 2.56; 95% CI: 0.53–12.43; *p* = 0.27), whereas in females the association was stronger and statistically significant (OR = 12.25; 95% CI: 1.27–118.36; *p* = 0.02) (Table 3).

In the multivariable logistic regression analysis, allocation to the VR group remained significantly associated with treatment success (adjusted OR = 5.41; 95% CI: 1.58–21.96; *p* = 0.011) (Table 4).

Female gender showed a borderline association (adjusted OR = 3.52; 95% CI: 1.02–13.82; *p* = 0.055). Neither age (OR = 1.33; 95% CI: 0.85–2.18; *p* = 0.22) nor DAS score (OR = 0.68; 95% CI: 0.37–1.20; *p* = 0.19) were significantly associated with treatment success. Treatment duration by group and completion status is summarized in Table 5.

Post hoc power-based sample size estimation (two-sided α = 0.05) suggested that 35 participants per group would be required to detect a difference in treatment success rates of 78.6% versus 46.4% with 80% power; with an assumed 10% dropout rate, this increases to 39 participants per group (78 total). For 90% power, 46 participants per group (92 total) would be required.

## 4. Discussion

This study evaluated the effectiveness of a VR-based distraction approach compared with a cartoon monitor in a cohort of individuals with mild ID during dental treatment. Moderate dental anxiety was used exclusively as an inclusion criterion to obtain a homogeneous study population. The results showed a higher treatment completion rate in the VR group compared with the monitor-based distraction group (78.6% vs. 46.4%), suggesting that VR may represent a useful supportive tool to facilitate dental care in this special care pediatric population. This likely reflects the greater immersive and engaging nature of VR compared to traditional monitor-based distraction. We underscore that, given the challenges typically encountered in managing dental anxiety among patients with NDDs, even a moderate increase in procedural success can significantly improve access to routine dental care. Conversely, treatment failure observed in both groups was primarily due to patient distress, uncooperative behavior, or an inability to complete the dental procedure within the allocated time. Specifically, in the monitor group, most failures were related to behavioral resistance, while in the VR group, failures mainly involved partial cooperation with persistent discomfort despite distraction.

Distraction techniques can be broadly categorized into active and passive approaches. As documented in previous studies, no significant differences were observed in children’s self-reported procedural pain between active and passive distraction methods, particularly with respect to anxiety-related outcomes [24,25]. In the present study, virtual reality was implemented as a passive immersive distraction method, providing emotionally engaging audiovisual stimulation without requiring active participation from the child. In fact, the VR experience consisted solely of immersive audiovisual stimulation aimed at sustaining attention and engagement. Consistent with previous work evaluating the effects of eight-dimensional audio analgesia and virtual reality environments, both approaches were found to be effective in modulating pain perception during restorative dental treatment in children with dental anxiety [26]. Audio analgesia approaches paved the way for the development of electronic device-based distraction techniques [27]. As previously reported [22], a VR-based approach can significantly enhance the effectiveness of dental treatment in individuals with NDDs, particularly those with ASD. Conventional approaches, in contrast, often leave individuals with NDDs uncomfortable and uncooperative during dental care [28]. The results of this study are consistent with previous findings suggesting the potential benefits of VR approaches compared to audio-based interventions in patients with mild ID [29]. In our study, however, instead of an audio-based treatment, participants in the control group received a monitor-based intervention, which involved watching a cartoon or video of their choice on a screen positioned in the modified dental operatory. It is worth noting that, although the selected cartoons were suitable for the children examined, they did not provide sufficient relaxation. As a result, the children were not fully at ease during dental treatment and remained outside their comfort zone. As previously suggested [29], and further supported by our findings, VR may be more effective than conventional distraction methods because it offers an enjoyable and fully immersive environment while simultaneously engaging higher-order cognitive and emotional processes.

In the stratified analysis, VR appeared to be particularly effective in female participants, with a markedly higher success rate compared to females in the monitor group. By contrast, the effect in males, although favorable, did not reach statistical significance. Several explanations may be considered. Gender differences in the expression and regulation of dental anxiety have been reported in previous studies, with females generally showing higher baseline anxiety and being more dentally fearful than males [30,31]. Nevertheless, they often respond more positively to supportive or distraction-based interventions. We posit that the positive response to VR treatment observed among females may arise from consistent gender-related differences in communication and coping behaviors, as highlighted by previous studies [32]. As further documented in the literature, female individuals with NDDs tend to exhibit significantly better social interaction and communication skills than males [33]. These psychosocial dynamics may partly explain the higher rate of treatment success observed among female participants in this study. Although gender differences in dental anxiety have been outlined in previous studies, the apparent gender effect on treatment success observed in our experiment should be interpreted cautiously and regarded as hypothesis-generating rather than conclusive.

This comparative observational study indicates that VR may improve treatment success in children with mild intellectual disability compared with a conventional distraction method. The primary objective of our study was to achieve treatment success in a special care population, rather than to reduce dental anxiety in individuals with NDDs. Moderate dental anxiety, assessed using the Dental Anxiety Scale (DAS), was used solely as an inclusion criterion. Nonetheless, several limitations should be considered. First, the study was conducted in a single specialized center with a relatively small sample size of 56 participants, which limits the generalizability of the findings and reduces the statistical power of subgroup analyses, particularly those stratified by gender. A post hoc power-based sample size estimation indicated that a larger sample would be required to confirm the observed effect with adequate statistical power. Based on the treatment success rates observed in this study (78.6% in the virtual reality group vs. 46.4% in the monitor group), approximately 35 participants per group would be needed to achieve 80% power, increasing to 39 per group when accounting for potential dropout. Therefore, although the present findings demonstrate a statistically significant difference between groups, the limited sample size suggests that these results should be interpreted cautiously and considered hypothesis-generating. Larger, adequately powered prospective studies are warranted to confirm the magnitude of the observed effect and to further explore subgroup differences. In addition, all treatments were performed in the same dental clinic by the same operators, ensuring methodological consistency but also potentially limiting external validity. Another potential limitation is related to the treatment completion rates which were assessed only in the short term after the intervention, focusing on completion of a single dental session; therefore, no conclusions can be drawn regarding the long-term effects of VR on dental anxiety or cooperation across repeated visits. Furthermore, the study population consisted exclusively of children aged 11–15 years with mild intellectual disability, and the results may not be extrapolated to younger children, adults, or patients with more severe forms of neurodevelopmental disorders. Notably, as previously documented, age may play a crucial role in dental anxiety, with younger individuals generally exhibiting higher levels of fear compared to older patients [34]. Another potential limitation is represented by the outcome definition of “treatment success” as completion within 50 min. In fact, this binary outcome does not capture other relevant dimensions such as behavioral cooperation, anxiety levels, or physiological stress responses, physiological stress markers, or patient-reported comfort. For this purpose, the Frankl Behavior Rating Scale would have been helpful in categorizing children’s behavior during and after dental. Another limitation of this study concerns the potential risk of observer bias, as the same clinician performed the dental procedures and evaluated treatment success. Nevertheless, participants were blinded to the study hypotheses, and the assessor was not informed about the study aims, which may have partially mitigated this potential source of bias. The assessor’s role was limited to determining whether the dental treatment was completed, without performing behavioral or engagement assessments.

Furthermore, individual variability in engagement was not formally assessed. Since children with mild ID may vary significantly in their cognitive and emotional response to immersive stimuli, the degree of distraction achieved likely differed across participants, acting as a potential confounding variable. Additionally, no assessment of patient preference or prior familiarity with digital technologies was included. Finally, individual variability in engagement with VR content may have influenced completion rate, and no assessment of patient preference or prior familiarity with digital technologies was included. In fact, we do not have detailed information regarding the patients’ previous exposure to digital devices. We emphasize that neither the DAS nor any additional questionnaire was administered after the dental restoration procedure, which represents a potential limitation of the study. A further limitation of this study is the non-randomized allocation of participants, as group assignment was determined by device availability and clinical scheduling. This pragmatic approach may introduce potential selection bias and therefore limits the ability to draw causal inferences from the observed findings.

We underscore that future studies with larger and more diverse samples, conducted across multiple centers and including longer follow-up, are warranted to confirm and extend these findings. We acknowledge that the relatively small sample size may have limited the overall statistical power, thereby increasing the potential risk of Type II error.

To mitigate these biases in future research, we recommend the use of independent, blinded evaluators to score patient behavior via video recordings without knowledge of the intervention group. Moreover, incorporating objective physiological stress markers—such as heart rate variability or galvanic skin response—alongside validated behavioral scales, would provide a more impartial and multi-dimensional assessment of anxiety reduction, independent of clinical observation. Incorporating more refined outcome measures during or at the end of the treatment, such as validated behavioral scales, physiological indicators, and patient-reported outcomes, would provide a more comprehensive understanding of VR’s impact on dental anxiety. In addition, further studies are warranted for investigating whether specific types of VR content (e.g., interactive games, relaxation environments) are more effective and evaluating the cost-effectiveness and feasibility of implementing VR in routine dental practice.

## 5. Conclusions

The immersive and emotionally engaging nature of VR likely contributes to improved cooperation and sustained engagement during dental procedures. Although a stronger effect was observed among female participants, this finding should be interpreted cautiously due to the limited sample size. Overall, VR represents a promising non-pharmacological tool for facilitating dental treatment in vulnerable pediatric populations, including patients with moderate dental anxiety. This preliminary work lays the foundation for future multicenter studies with larger samples and longer follow-up periods to confirm these results and to explore potential gender- and age-specific responses to VR-based interventions. This study aimed to extend existing evidence by evaluating whether an immersive, passive virtual reality distraction approach could facilitate dental treatment completion in a special care pediatric population. We emphasize that the cost-effectiveness and feasibility of implementing VR in routine dental practice should be carefully considered by clinicians, particularly in light of the higher treatment completion rates observed among individuals with NDDs.

## Figures and Tables

**Table 1:** Baseline characteristics of study participants by group. Mean age and mean dental anxiety scores (DAS) with standard deviations are reported for children assigned to the monitor group (n = 28) and the VR group (n = 28).

Group	Number	Age	DAS
Monitor	28	12.9 (±1.51)	10.4 (±1.1)
VR	28	12.9 (±1.27)	10.5 (±1.17)

**Table 2:** Treatment outcomes by group. Numbers and percentages of successful and failed dental treatments are shown for the VR and monitor distraction groups. Odds ratios (ORs) and risk ratios (RRs) with 95% confidence intervals (CIs) are reported, with the monitor group serving as reference.

Group	Success n (%)	Failure n (%)	OR (95% CI)	RR (95% CI)	*p*-Value ^a^
VR	22 (78.6)	6 (21.4)	4.12 (1.16–16.47)	2.50 (1.14–5.50)	0.026
Monitor	13 (46.4)	15 (53.6)	Reference	Reference	-

^a^ Comparisons between groups (VR vs. Monitor) were performed using the Chi-square test with Fisher’s exact test.

**Table 3:** Treatment outcomes stratified by gender. Numbers and percentages of successful and failed dental treatments are shown for males and females. Odds ratios (ORs) with 95% confidence intervals (CIs) are reported for VR versus monitor distraction within each gender.

Group	Success n (%)	Failure n (%)	OR (95% CI)	*p*-Value *
Males (n = 26)	13 (50.0)	13 (50.0)	2.56 (0.53–12.43)	0.27
Females (n = 30)	21 (70.0)	9 (30.0)	12.25 (1.27–118.36)	0.02

* Comparisons between groups (VR vs. Monitor) were performed using the Chi-square test with Fisher’s exact test. No significant differences were found in baseline DAS scores between participants who completed treatment and those who did not (mean 10.3 vs. 10.7; *p* = 0.25).

**Table 4:** Multivariable logistic regression for predictors of treatment success. Adjusted odds ratios (ORs) with 95% confidence intervals (CIs) and *p*-values are reported. The model included group assignment (VR vs. monitor), age, gender, and baseline dental anxiety (DAS score) as covariates.

Group	OR	95% CI	*p*-Value *
VR vs. Monitor	5.41	1.58–21.96	0.0108
Age (years)	1.33	0.85–2.18	0.2236
Female vs. Male	3.52	1.02–13.82	0.0551
DAS score	0.68	0.37–1.20	0.1917

* Comparisons between groups (VR vs. Monitor) were performed using the Chi-square test with Fisher’s exact test; the Mantel–Haenszel method was applied to estimate the common OR stratified by gender; Differences in DAS scores between successful and unsuccessful treatments were evaluated using Student’s *t*-test.

**Table 5:** Descriptive statistics for the duration of the dental restoration procedure. Data are presented as mean ± standard deviation (SD), median, minimum, and maximum values. Comparisons were conducted between treatment groups (Monitor vs. VR) and between outcome categories (Completed vs. Not completed).

Section	Level	Mean ± SD	Median	Min	Max	*p*-Value
Group	Monitor (n = 28)	27.89 ± 6.32	29	15	40	0.0946
	VR (n = 28)	31.36 ± 8.70	33	0	41	0.0946
Outcome	Not completed (n = 21)	23.05 ± 7.45	25	0	32	0.00000241
	Completed (n = 35)	33.57 ± 4.62	33	25	41	0.00000241

## Data Availability

The original contributions presented in this study are included in the article. Further inquiries can be directed to the corresponding author.

## Abbreviations

The following abbreviations are used in this manuscript:

ASD	Autism spectrum disorder
BIF	Borderline intellectual functioning
CI	Confidence interval
DAS	Dental anxiety scale
ID	Intellectual disability
MA	Moderate anxiety
NA	No anxiety
NDD	Neurodevelopmental disorder
OR	Odds ratio
RR	Risk ratio
SA	Severe anxiety
SLA	Slight anxiety
VR	Virtual reality
